# Readiness for Change in the Implementation of a 3D Printing Initiative in a Catalan Tertiary Hospital Using the Normalization Process Theory: Survey Study

**DOI:** 10.2196/47390

**Published:** 2023-10-06

**Authors:** Francesc López Seguí, Joan Cos Codina, Laura Ricou Ríos, María Isabel Martínez Segura, Laura Miró Mezquita, Raquel Escrich Navarro, Meritxell Davins Riu, Oriol Estrada Cuxart, German Anashkin Kachalin, Daniel Moreno-Martínez

**Affiliations:** 1 Research Group on Innovation, Health Economics and Digital Transformation Institut Germans Trias i Pujol Badalona Spain; 2 Hospital Germans Trias i Pujol Institut Català de la Salut Badalona Spain; 3 Chair in ICT and Health Centre for Health and Social Care Research University of Vic - Central University of Catalonia Vic Spain; 4 Faculty of Health Sciences Universitat Oberta de Catalunya Barcelona Spain

**Keywords:** change management, normalization process theory, NPT, 3D printing, readiness for change, Normalization Measure Development questionnaire, NoMAD, implementation, tertiary hospital, barrier, readiness, printing, survey, development, questionnaire, support, communicate, assessment, users, transformation

## Abstract

**Background:**

The high failure rate of innovation projects motivates us to understand the perceptions about resistances and barriers of the main stakeholders to improving success rates.

**Objective:**

This study aims to analyze the readiness for change in the implementation of a 3D printing project in a Catalan tertiary hospital prior to its implementation.

**Methods:**

We used a web-based, voluntary, and anonymous survey using the Normalization Measurement Development questionnaire (NoMAD) to gather views and perceptions from a selected group of health care professionals at Germans Trias i Pujol University Hospital.

**Results:**

In this study, 58 professionals, including heads of service (n=30, 51%), doctors (n=18, 31%), nurses (n=7, 12%), and support staff (n=3, 5%), responded to the questionnaire. All groups saw the value of the project and were willing to enroll and support it. Respondents reported the highest scores (out of 5) in cognitive participation (mean 4.45, SD 0.04), coherence (mean 3.72, SD 0.13), and reflective monitoring (mean 3.80, SD 0.25). The weakest score was in collective action (mean 3.52, SD 0.12). There were no statistically significant differences in scores among professions in the survey.

**Conclusions:**

The 3D printing project implementation should pay attention to preparing, defining, sharing, and supporting the operational work involved in its use and implementation. It should also understand, assess, and communicate the ways in which the new set of practices can affect the users and others around them. We suggest that health officers and politicians consider this experience as a solid ground toward the development of a more efficient health innovation system and as a catalyst for transformation.

## Introduction

The largest hindrance for innovation projects is the high failure rate [1,2]. In the public sector, studies suggest that just 15% of innovation projects are fully successful, while 50% are successful in the health care sector [3]. It appears that high failure rates are consistent over time. As such, it seems necessary to understand the perceptions about resistances and barriers of the main stakeholders to improving success rates.

The 3D printing (3DP) process involves generating a 3D solid object from a digital model. It is one of the disruptive technologies that has the potential to substantially transform health care and represents a big opportunity for medical organizations. The applications of 3DP in the medical and clinical fields are diverse, including personalized presurgical treatment and preoperative planning, customized surgical tools and prostheses, testing of different devices in specific pathways, improving medical and patient education, bioprinting of implantable tissues, and personalized 3D drug printing, among others [4].

Normalization process theory (NPT) is a sociological toolkit used to understand the dynamics of implementing, embedding, and integrating new technologies or complex interventions [5]. It focuses on the processes leading to new practices becoming embedded in everyday work (ie, what makes an innovation project become accepted, used, and successful vs rejected). NPT identifies 4 constructs, namely, coherence, cognitive participation, collective action, and reflexive monitoring, to classify the “human work” around a new practice. Each construct is divided into 4 subconstructs, thus providing 16 checking points for success or failure. NPT has been effectively used to help intervention development and implementation planning as well as for evaluating and understanding implementation processes themselves, offering a valuable set of conceptual tools to aid in the understanding of implementation as a dynamic process [6].

NPT was conceived to understand and support innovation processes in the health care sector. It applies to new and emergent situations and complicated interventions, such as new working processes or the implementation of new technologies. In the context of technological innovation, NPT has largely been used by researchers and practitioners in hospitals and health care organizations in the fields of cardiology [7], telerehabilitation [8], patient-held health IT adoption [9], electronic health records [10], or web-based patient feedback [11]. A published systematic review presented results from studies using NPT as the primary approach for the collection, analysis, and reporting of data in studies in the health care sector, showing that it can effectively assist in the explanation of the success or failure of specific implementation projects [6]. NPT provides a conceptual vocabulary for rigorous studies of implementation processes and identifies, characterizes, and explains empirically identifiable mechanisms that motivate and shape implementation processes.

Previous research about the expectations of health care staff prior to the implementation of digital pathology (DIPA) by means of NPT in the form of semistructured interviews and the Normalization Measure Development (NoMAD) questionnaire was carried out [12,13]. Overall, the authors reported staff feeling sufficiently tech-savvy to be able to use DIPA, having high expectations as well as motivation and readiness for the upcoming changes. However, the employees were skeptical regarding the allocation of resources, and few had knowledge of the potential effects of DIPA. Based on the findings, it seems to be important not only to provide a thorough introduction to the new intervention and the changes it will entail, but also to continue to ensure that the staff know how it works and why it is necessary to implement it. Other studies have explored key stakeholders’ perceptions of the facilitators and barriers to implementing electronic systems for medicine management in hospital settings using semistructured interviews with NPT as the theoretical framework. They concluded that enhanced patient safety and efficiency in health care delivery emerged as key facilitators to system implementation, as well as the need to have clinical champions and a multidisciplinary implementation team to promote engagement and cognitive participation. Key barriers included inadequate training and organizational support and the need for ease and confidence in system use to achieve collective action. Many themes that are potentially transferable to other national settings have been identified and extend the evidence base [14].

In this context, this study aims to analyze the readiness for change in the implementation of a 3DP facility in a Catalan tertiary hospital prior to its implementation using the NPT as a background theory and the NoMAD as a validated instrument. The main objective of this work is to identify the perceptions of the different groups of professionals about the implementation of a 3DP facility. As secondary objectives, we aim to assess the use of the NoMAD as a tool for analysis, identify action areas that can improve the implementation, define a system or methodological model that can be used in future innovation initiatives, and provide fundamentals for the use of management tools in a public health system.

## Methods

### Data Collection

A web-based, voluntary, and anonymous survey was sent using Microsoft Forms to the Germans Trias i Pujol University Hospital board of directors and potential users of the 3DP project (Multimedia Appendix 1). At the same time, the recipients were invited to forward the survey to whoever they thought might be involved with the project. We received 99 responses, of which 41 were excluded because the respondent considered that they would not be involved in the 3DP project. The remaining data set of 58 complete responses was analyzed. No information that could identify the respondents was registered in the survey.

Respondents were classified into 1 of 2 groups according to the type of role they will have in the 3DP project (management and supervision roles or utilization roles) and by professional groups (doctors, heads of service, nurses, or support staff). Information on their age was also recorded. Each response to the core 20 items of the questionnaire (Multimedia Appendix 1) comprised a numerical answer quantifying their level of agreement with each statement. Then, the mean value of each group was computed. Differences were assessed using ANOVA, with *P*<.05 considered significant. Data analysis was performed using Python (Python Software Foundation) and Jupyter Notebook (Fernando Pérez). Petal charts were obtained using the plotly library.

### NPT Core Constructs

NPT is an action theory, which means it is concerned with explaining what people do rather than their attitudes or beliefs. We divided action according to the 4 NPT constructs that represent different kinds of work that people would do to implement the 3DP project, namely, coherence, cognitive participation, collective action, and reflexive monitoring [15]. The questionnaire comprised 20 core questions. Each construct represented a generative mechanism of social action (ie, different kinds of work that people do as they work around a set of practices of the project). Further explanation on the 4 NPT constructs can be found in Multimedia Appendix 2.

### Ethical Approval

Ethical review and approval were obtained from Germans Trias Research Institute Ethics (PI-22-072) on March 25, 2022. Informed consent was obtained from all subjects involved in the study.

## Results

### General Overview

A data set comprising 58 responses was used in the analysis. The respondents’ descriptions showed that they had ≥11 years of experience (n=34, 58%) and corresponded to the professional profiles of heads of service (n=30, 51%), doctors (n=18, 31%), nurses (n=7, 12%), and support staff (n=3, 5%). We thus had a mean profile of a senior hospital leader. Respondents reported the highest scores (out of 5) in cognitive participation (mean 4.45, SD 0.04), coherence (mean 3.72, SD 0.12), and reflective monitoring (mean 3.80, SD 0.25). The weakest score was in collective action (mean 3.52, SD 0.12).

### Analysis by Construct and Role in the Project

#### Coherence

**Figure 1:**
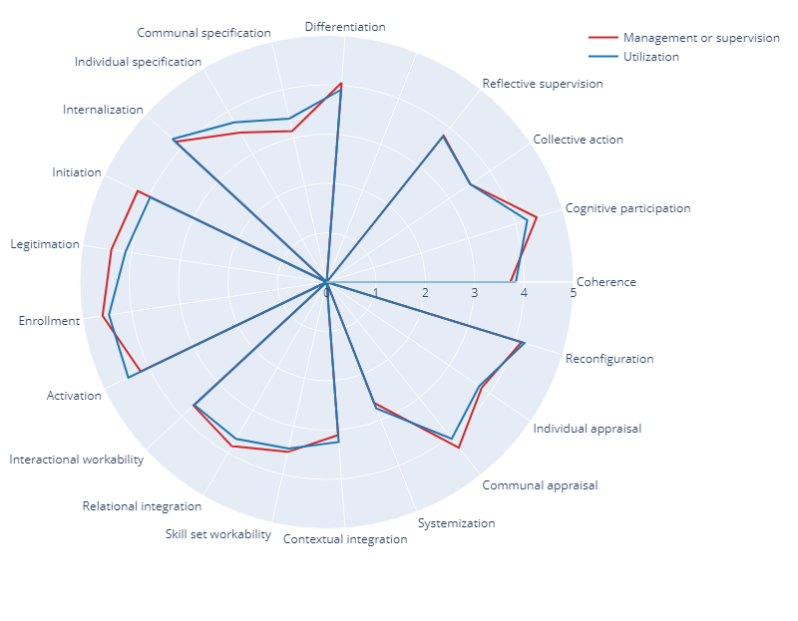
Results according to the role the respondent will have in the 3D project.

There were no major or statistically significant differences in scores between management and supervision and utilization roles in the survey. Differences were analyzed when deemed convenient (Figure 1 and Table 1).

**Table 1:** Scores according to the role the respondents will have in the 3D printing project.

			Management and supervision, mean (SD)		Direct utilization, mean (SD)		Total, mean (SD)		*P* value
**Coherence**			3.72 (0.13)		3.83 (0.21)		3.77 (0.12)		N/A^a^
	I can see how this project differs from usual ways of working	4.05 (0.84)		3.91 (0.82)		3.98 (0.29)		.56	
	Staff in the organization have a shared understanding of the purpose of the project	3.14 (1.13)		3.40 (1.09)		3.27 (0.79)		.38	
	I understand how this project affects the nature of my own work	3.50 (0.96)		3.74 (0.70)		3.62 (0.58)		.28	
	I can see the potential value of the project for my work	4.18 (0.85)		4.26 (0.61)		4.22 (0.52)		.70	
**Cognitive participation**			4.45 (0.04)		4.25 (0.12)		4.35 (0.05)		N/A
	There are key people who drive the project forward and get others involved	4.25 (0.55)		3.97 (0.76)		4.11 (0.41)		.16	
	I believe that participating in the project is a legitimate part of my role	4.41 (0.59)		4.12 (0.64)		4.27 (0.44)		.09	
	I’m open to working with colleagues in new ways to use the project.	4.55 (0.51)		4.46 (0.51)		4.50 (0.36)		.52	
	I will continue to support the project	4.59 (0.50)		4.46 (0.51)		4.53 (0.36)		.33	
**Collective action**			3.52 (0.12)		3.49 (0.12)		3.50 (0.09)		N/A
	I can easily integrate the project into my existing work	3.68 (0.95)		3.66 (0.91)		3.67 (0.66)		.92	
	The project disrupts working relationships	3.48 (0.98)		3.34 (1.03)		3.41 (0.71)		.63	
	I have confidence in other people’s ability to use the project	4.19 (0.87)		4.00 (0.82)		4.10 (0.60)		.42	
	Work is assigned to those with skills appropriate to the project.	4.11 (0.88)		3.91 (0.64)		4.01 (0.54)		.35	
	Sufficient training is provided to enable staff to use the project.	2.94 (1.21)		3.00 (0.91)		2.97 (0.75)		.86	
	Sufficient resources are available to support the project.	2.47 (1.12)		2.85 (0.95)		2.66 (0.73)		.22	
	Management adequately supports the project	3.75 (1.02)		3.65 (0.91)		3.70 (0.68)		.70	
**Reflective monitoring**			3.80 (0.25)		3.78 (0.15)		3.79 (0.14)		N/A
	I am aware of reports about the effects of the project	2.63 (1.16)		2.75 (0.88)		2.69 (0.72)		.68	
	I value the effects the project has had on my work	4.29 (0.72)		4.06 (0.48)		4.18 (0.43)		.16	
	The staff agree that the project is worthwhile	3.80 (0.95)		3.74 (0.68)		3.77 (0.58)		.80	
	Feedback about the project can be used to improve	4.50 (0.51)		4.34 (0.54)		4.42 (0.37)		.28	
	I can modify how I work with the project	3.76 (1.00)		4.03 (0.68)		3.90 (0.60)		.25	

^a^N/A: not applicable.

With regard to the sense-making work that people do when they are faced with the problem of operationalizing the set of practices involved in the implementation of 3DP, the average score of all 4 subconstructs was 3.77 (SD 0.12), with scores of 3.83 (SD 0.21) from respondents with utilization roles and 3.72 (SD 0.13) from those with management and supervision roles. The span between subconstructs (both roles together) was 0.95. The span between top and bottom scores (roles split) was 1.12. Communal specification received the lowest score (mean 3.27, SD 0.79; mean 3.4, SD 0.70 and mean 3.14, SD 1.13 from respondents with utilization and management and supervision roles, respectively). Internalization received a higher score (mean 4.22, SD 0.52; mean 4.26, SD 0.61 and mean 4.18, SD 0.85 from respondents with utilization and management and supervision roles, respectively).

#### Cognitive Participation

With regard to the relational work that people do to build and sustain a community of practice around 3DP, the average score of all 4 subconstructs was 4.35 (SD 0.05), with scores of 4.25 (SD 0.12) from respondents with direct utilization roles and 4.45 (SD 0.04) from those with management and supervision roles. This was the construct that received the highest score (mean 4.53, SD 0.36; roles together) and the highest bottom score (mean 4.11, SD 0.41). The span between subconstructs (both roles together) was 0.42, which was the lowest in its category. The span between top and bottom scores (roles split) was 0.62.

Initiation received the lowest score (mean 4.11, SD 0.41; mean 4.25, SD 0.55 and mean 3.97, SD 0.76 from respondents with management and supervision and utilization roles, respectively), while activation received a higher score (mean 4.53, SD 0.36; mean 4.46, SD 0.51 and mean 4.59, SD 0.50 from respondents with utilization and management and supervision roles, respectively).

#### Collective Action

With regard to the operational work that people do to enact the set of practices involved in the implementation of 3DP, the average score of all 4 subconstructs was 3.50 (SD 0.09), with scores of 3.49 (SD 0.12) from respondents with direct utilization roles and 3.52 (SD 0.12) from those with management and supervision roles. For both roles together, this construct received the lowest score, with the span between subconstructs being 0.58.

Contextual integration received an average score of 4.01 (SD 1.21; mean 4.11, SD 0.97 and mean 3.91, SD 0.75 from respondents with utilization and management and supervision roles, respectively). The question receiving the lowest score of 2.66 (SD 0.73; roles together) was “Sufficient resources are available to support 3DP,” with respondents with management and supervision roles scoring slightly lower (mean 2.47, SD 1.12) than those with utilization roles (mean 2.85, SD 0.95). Relational integration received a higher score (mean 3.76, SD 0.91; mean 3.67, SD 0.68 and mean 3.84, SD 1.02 from respondents with utilization and management and supervision roles, respectively).

#### Reflexive Monitoring

With regard to the appraisal work that people do to assess and understand the ways in which 3DP may affect them and others around them, the average score of all 4 subconstructs was 3.79 (SD 0.15), with scores of 3.78 (SD 0.14) from respondents with direct utilization roles and 3.8 (SD 0.25) from those with management and supervision roles. The span between subconstructs (both roles together) was 1.49. The span between top and bottom scores (roles split) was 1.66, which was the highest recorded and may be relevant when reaching conclusions.

Systemization received the lowest score (mean 2.69, SD 1.16; mean 2.75, SD 0.88 and mean 2.63, SD 0.72 from respondents with utilization and management and supervision roles, respectively). The question receiving the lowest score of 2.63 (SD 1.16; roles together) was, “I am aware of reports about the effects of 3DP”.

### Analysis by Construct and Professional Group

**Figure 2:**
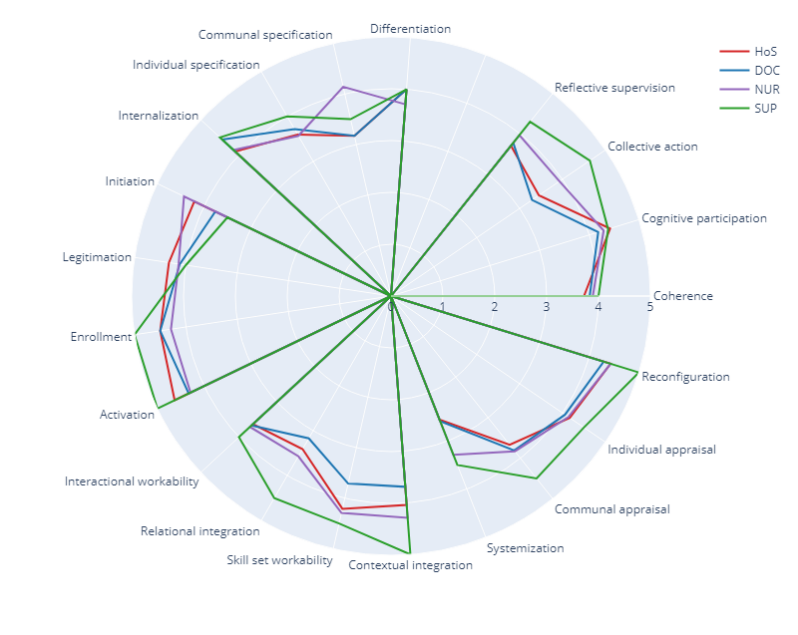
Results according to the professional group the respondent will belong to in the 3D project. DOC: doctors; HoS: head of service; NUR: nurses; SUP: support staff.

All professional groups tended to follow the same scoring pattern, except where commented, and scores between all groups did not show large differences. Scores were very similar in the coherence and cognitive participation constructs, and the largest span between groups’ scores was 0.24 in cognitive participation (Figure 2 and Table 2).

**Table 2:** Scores according to the respondents’ professional group.

		HoS^a^, mean (SD)	DOC^b^, mean (SD)	NUR^c^, mean (SD)	SUP^d^, mean (SD)	Total, mean (SD)	*P* value
**Coherence**		3.72 (0.16)	3.83 (0.19)	3.89 (0.37)	4.00 (0.91)	3.86 (0.20)	N/A^e^
	I can see how this project differs from usual ways of working	4.00 (0.86)	4.00 (0.61)	3.71 (1.25)	4.00 (1.41)	3.93 (0.51)	.88
	Staff in the organization have a shared understanding of the purpose of the project	3.17 (1.17)	3.17 (0.95)	4.14 (0.38)	3.50 (2.12)	3.50 (0.58)	.87
	I understand how this project affects the nature of my own work	3.60 (0.88)	3.72 (0.69)	3.57 (0.98)	4.00 (0)	3.72 (0.32)	.18
	I can see the potential value of the project for my work	4.10 (0.81)	4.44 (0.51)	4.14 (0.69)	4.50 (0.71)	4.30 (0.33)	.39
**Cognitive participation**		4.42 (0.10)	4.18 (0.17)	4.29 (0.06)	4.38 (0.36)	4.31 (0.09)	N/A
	There are key people who drive the project forward and get others involved	4.21 (0.56)	3.76 (0.86)	4.43 (0.53)	3.50 (0.71)	3.97 (0.34)	.05
	I believe that participating in the project is a legitimate part of my role	4.33 (0.72)	4.12 (0.62)	4.14 (0.38)	4.00 (0)	4.15 (0.24)	.64
	I’m open to working with colleagues in new ways to use the project.	4.50 (0.51)	4.50 (0.51)	4.29 (0.49)	5.00 (0)	4.57 (0.19)	.37
	I will continue to support the project	4.63 (0.49)	4.33 (0.49)	4.29 (0.49)	5.00 (0)	4.56 (0.18)	.06
**Collective action**		3.45 (0.12)	3.29 (0.22)	3.93 (0.26)	4.64 (0.36)	3.83 (0.12)	N/A
	I can easily integrate the project into my existing work	3.63 (0.98)	3.67 (1.00)	3.71 (0.76)	4.00 (0)	3.75 (0.34)	.96
	The project disrupts working relationships	3.41 (0.97)	3.17 (1.05)	3.57 (0.79)	4.50 (0.71)	3.66 (0.44)	.32
	I have confidence in other people’s ability to use the project	4.21 (0.71)	3.71 (1.06)	4.29 (0.49)	4.50 (0.71)	4.18 (0.37)	.17
	Work is assigned to those with skills appropriate to the project.	4.04 (0.86)	3.69 (0.46)	4.29 (0.49)	5.00 (0)	4.26 (0.23)	.12
	Sufficient training is provided to enable staff to use the project.	2.70 (1.09)	2.82 (0.81)	4.00 (0)	5.00 (0)	3.63 (0.24)	<.001
	Sufficient resources are available to support the project.	2.46 (1.01)	2.53 (0.92)	3.67 (0.52)	5.00 (0)	3.42 (0.31)	<.001
	Management adequately supports the project	3.68 (0.97)	3.47 (1.06)	4.00 (0.63)	4.50 (0.71)	3.91 (0.42)	.41
**Reflexive monitoring**		3.71 (0.22)	3.78 (0.19)	3.97 (0.16)	4.30 (0.39)	3.94 (0.12)	N/A
	I am aware of reports about the effects of the project	2.56 (1.06)	2.59 (0.96)	3.29 (0.76)	3.50 (0.71)	2.99 (0.39)	.21
	I value the effects the project has had on my work	4.17 (0.74)	4.06 (0.43)	4.14 (0.38)	4.50 (0.71)	4.22 (0.28)	.76
	The staff agree that the project is worthwhile	3.67 (0.95)	3.81 (0.66)	3.83 (0.41)	4.50 (0.71)	3.95 (0.35)	.53
	Feedback about the project can be used to improve	4.43 (0.51)	4.28 (0.59)	4.43 (0.53)	5.00 (0)	4.54 (0.20)	.31
	I can modify how I work with the project	3.71 (0.94)	4.18 (0.72)	4.14 (0.38)	4.00 (0)	4.01 (0.26)	.27

^a^HoS: head of service.

^b^DOC: doctor.

^c^NUR: nurse.

^d^SUP: support staff.

^e^N/A: not applicable.

Support staff scored all constructs higher than all other groups, scoring 0.34 above the median of all 4 groups and 0.56 higher than the lowest-scoring group (doctors). They scored remarkably higher in collective action and reflexive monitoring (0.81 and 0.36 above the median of all 4 groups, respectively). The lowest scores given by support staff (3.50) were in the subconstructs communal appraisal (coherence), initiation (cognitive participation), and systemization (reflexive monitoring). Nursing professionals scored the subconstruct communal specification (coherence) remarkably higher (mean 4.14, SD 0.38) than other groups and gave the lowest scores in systemization (mean 3.29, SD 0.76), individual specification and relational integration (mean 3.57, SD 0.79), and communal appraisal (mean 3.83, SD 0.41).

Doctors and heads of units gave high scores in several subconstructs, in parallel with the other groups. They did not score remarkably high in any construct or subconstruct. Instead, they scored remarkably low in contextual integration (mean 2.91, SD 3.16 and mean 3.07, SD 2.41 for doctors and heads of service, respectively) and in the question relating to the availability of sufficient resources (mean 2.53, SD 0.92 and mean 2.46, SD 1.01, respectively), which was the lowest scoring of all subconstructs. They scored lower than the other groups in systemization (mean 2.59, SD 0.96 and mean 2.56, SD 1.06, respectively), contextual integration (mean 2.91, SD 3.16 and mean 3.07, SD 2.41, respectively), communal specification (mean 3.17, SD 0.95 and mean 3.17, SD 1.17, respectively), and skill set workability (mean 2.82, SD 0.81 and mean 2.70, SD 1.09, respectively). They scored significantly lower than nurses and support staff in collective action, which was the construct with the largest span between the lowest and the highest scores (1.35). In contextual integration (collective action), there were important differences between all groups, showing that perceptions are not aligned.

### Analysis of Perceptions About Integration by Role in the Project

Respondents feel familiar with 3DP being a normal part of their work (mean 3.11, SD 1.92) and using it in their daily work (mean 3.62, SD 1.48), but they don’t think it will become a normal part of their work (mean 2.37, SD 1.84). Analyzing by roles or professional groups do not significantly alter the conclusions (Tables 3 and 4).

**Table 3:** Scores according to the respondents’ role.

	Management and supervision, mean (SD)	Direct utilization, mean (SD)	Total, mean (SD)	*P* value
When you use or imagine using 3DP^a^ in your daily work, how familiar does it feel?	3.04 (3.12)	3.18 (2.29)	3.11 (1.92)	.71
Do you feel that 3DP is a normal part of your work?	2.04 (2.99)	2.69 (2.19)	2.37 (1.84)	.06
Do you feel 3DP will become a normal part of your work?	3.54 (2.54)	3.69 (1.64)	3.62 (1.48)	.59

^a^3DP: 3D printing.

**Table 4:** Scores according to the respondents’ professional group.

	HoS^a^, mean (SD)	DOC^b^, mean (SD)	NUR^c^, mean (SD)	SUP^d^, mean (SD)	Total, mean (SD)	*P* value
When you use or imagine using 3DP^e^ in you daily work, how familiar does it feel?	2.91 (3.16)	3.36 (1.79)	3.07 (2.41)	4.00 (1.73)	3.33 (1.14)	.45
Do you feel that 3DP is a normal part of your work?	2.26 (2.92)	2.80 (2.12)	2.64 (2.50)	1.66 (2.52)	2.34 (1.26)	.36
Do you feel 3DP will become a normal part of your work?	3.63 (2.34)	3.80 (1.50)	3.21 (1.90)	3.66 (2.52)	3.58 (1.03)	.64

^a^HoS: head of service.

^b^DOC: doctor.

^c^NUR: nurse.

^d^SUP: support staff.

^e^3DP: 3D printing.

## Discussion

### Principal Findings

Having sound knowledge of the different perceptions and concerns of the stakeholders of a new solution in health care, such as a 3DP facility, is a valuable stepping stone toward designing and deploying highly effective actions, especially if they are specific to each group and follow the tools and best practices of organizational change management (OCM) [1,2]; this refers to diagnosing and designing strategies and actions toward improving the level of acceptance, use, and integration of changes in organizations. In this context, NPT and the NoMAD have proved to be useful tools for this purpose [16].

Survey respondents had a sound perception of the community of practice around 3DP (ie, legitimation, organization, and action toward its implementation); however, the operational side of making 3DP work in practice and what needed to be done to make it happen scored weakly, with the lack of resources and skills being the largest concern. There was also a concern about the practices, artefacts, and other elements required. In terms of appraisals, respondents were highly concerned about reporting systems and how they assessed whether 3DP was worth the effort and what improvements could be made. When the scores provided by the different professional groups differed for a given subconstruct, it is important to consider actions that are specific to each group. This may be crucial for better acceptance, use, and integration of 3DP following the general theory, best practices, and specific tools available in OCM.

According to our findings, actions to improve the implementation and benefits of 3DP should engage the most relevant stakeholders in the design and definition of the project and its implementation; receive the direct, public, and active sponsorship of the most relevant management and medical positions in the hospital; communicate proactively in a segmented manner using customized contents and messages; and enable a management structure that includes a change manager as well as a few success indicators. As the design and deployment of change management plans usually implies practical difficulties, we suggest using methodological tools that can provide structure and simplification to the team involved in change management. As a suggestion, the implementation tool SIGS [17] is a middle-level methodology that helps to connect high-level concepts, recommendations, and ideas with practical and handy actions. SIGS proposes a sequence named “Stakeholders–Impact–Gap–Strategy” that creates a methodological path to create change management actions that are rooted in the specific needs of the stakeholders and is, therefore, results-oriented.

Our results are aligned with the conclusions reached by previous studies that used NPT to assess expectations prior to the implementation of DIPA and found that the participants “reported feeling sufficiently tech-savvy to be able to use DIPA” and had high expectations, motivation, and readiness for the upcoming changes [12]. However, the employees were skeptical regarding the allocation of resources, and few were aware of reports about the effects of DIPA. Based on the findings, it seems to be important to provide not only a thorough introduction to the new intervention and the changes it will entail, but also to continue to ensure that the staff know how it works and why it is necessary to implement.

We suggest that future innovation initiatives in the health care sector can improve their success rates substantially by following the steps carried out in this paper, namely, a study of the perception of stakeholders using NPT and the set of actions (or alike) that we suggested hereinbefore. This should be done at very early stages of the project, starting at its very inception phase, as prevention is the major factor for reducing resistance in any new implementation (as stated often in OCM literature).

The following is a sample of the actions that may be derived from this analysis: (1) running a workshop with the stakeholders to discuss the results from this research and collect further perceptions and suggestions for a better implementation from those who will be the users of 3DP; (2) creating a presentation of 3DP clarifying all the concerns that stakeholders have and customizing it to the different user groups; (3) running a meeting or workshop led by the sponsors (eg, chief executive officer or chief marketing officer) explaining the project and showing their direct support; (4) creating a change management office that gives support to the implementation of this project (and others) using specific OCM techniques and approaches; (5) agreeing on a change scoreboard with 4-6 indicators to assess success for the implementation of 3DP; (6) creating (or using, if existing) an innovation newsletter that reports regularly about the implementation, cases, and quick wins for 3DP (and other projects, if existing); and (7) creating a committee with stakeholders to follow up on implementation and usage until complete integration.

### Limitations

Several limitations should be considered. First, the fact that the project was not in place nor formally presented at the time of the survey may have influenced low scores in reflective monitoring and collective action, as these constructs refer to appraisal and operational work, respectively. This is a limitation inherent to a preimplementation analysis. Second, representativeness of the respondents is uncertain as it was not possible to identify the exact total number of potential users of 3DP in direct roles, such as management and supervision and utilization. The majority of respondents were heads of services, and thus the results should be understood in the context of this sample. Finally, further research is suggested using qualitative methods with the different professional groups involved to validate and deepen the results. This would contribute to the development of a more robust analysis in which new factors may emerge and enrich the awareness about distinctive perceptions of professionals.

### Conclusions

In this study about the readiness for change based on the expectations of the different users of a 3DP facility in a large hospital prior to its implementation, we learned that all groups of professionals involved see the value of the project and are willing to enroll and support it. Nevertheless, its implementation should pay attention to preparing, defining, sharing, and supporting the operational work involved in its use and implementation. It is also important to understand, assess, and communicate the ways in which the new set of practices may affect the users and others around them. We suggest that health officers and politicians consider this experience and its tools and framework in health care change management as a solid ground toward the development of a more efficient health innovation system and as a catalyst for transformation.

## Appendix

Multimedia Appendix 1 Survey (English version).

Multimedia Appendix 2 Normalization process theory core constructs.

## Abbreviations

3DP: 3D project
DIPA: digital pathology
NoMAD: Normalization Measure Development
NPT: normalization process theory
OCM: organizational change management

## Data Availability

Data from the study can be requested by contacting the corresponding author.
