# Supplementary material for: Readiness for Change in the Implementation of a 3D Printing Initiative in a Catalan Tertiary Hospital Using the Normalization Process Theory: Survey Study
Source: JMIR Hum Factors. 2023 Oct 6;10:e47390. doi: 10.2196/47390 (PMC10589830; doi:10.2196/47390)
Supplement: Multimedia Appendix 1 [file humanfactors_v10i1e47390_app1.pdf]

Multimedia Appendix 1: Survey (English version)

1. How many years have you been working at Germans Trias Hospital? (if you changed centers but were still part of the ICS, add up the total years)
   - Less than 1; 1-2; 3-5; 6-10; 11-15; More than 15

2. How would you describe your professional group?
   - Head of Service/Unit/Section/Department; Nursing Supervisor; Doctor (non-surgical); Doctor (surgical); Nurse; Researcher; Resident; Administrative Staff; Staff of Support Units; others

3. In which area of the Hospital do you work? [Open answer]

4. This questionnaire asks about the implementation of the 3D Program. We understand that some of the people involved may have different roles or even more than one. Choose the one you identify with the most. Please answer the rest of the survey from the perspective of this role you have selected.
   - I will be involved in the management or supervision of the 3D Program; I will be involved in the use of 3D technology, either 3D printing or image virtualization (operational use, claimant, or service user, etc.); I will not be directly involved in any role of management, supervision or use of the 3D Program; others

5. When you use (or imagine using) 3D technology in your daily professional life, how familiar is it to you? [1-5]

6. Do you feel that 3D technology is a normal part of your work at the Hospital? [1-5]

7. Do you feel that 3D technology will become a normal part of your work at the Hospital? [1-5]

8. Answer the following questions below: [1-5]
   - I can see/appreciate how this project differs from the usual ways of working
   - Organizational staff have a common understanding of the purpose of this project
   - I understand how this project affects the nature of my own work
   - I can see/appreciate the potential value of this project for my work
   - There are people who lead and push the project forward and get others involved
   - I believe that participating in the project is a legitimate part of my role
   - I am open to working with other colleagues on new ways of using the project
   - I will continue to support the project
   - I can easily integrate the project into my current work
   - The project modifies the current working relationships
   - I rely on other people's skill in using the project
   - Work is assigned to people who have the appropriate skills for the project
   - Sufficient training is given to enable staff to implement/use the project
   - Sufficient resources are available to support the project
   - The Management provides adequate support to the project
   - I am aware of reports on the effects/results of the project
   - Staff agree that the project is worthwhile
   - I appreciate (value) the effects that the project has had (or will have) on my work
   - Feedback on the project can be used for improvement (in the future)
   - I can change how I work with the project

9. Write here anything you would like to share: ideas and considerations you had when answering the survey, proposed actions to improve implementation, or any suggestions. (Open answer)
