# Supplementary material for: Readiness for Change in the Implementation of a 3D Printing Initiative in a Catalan Tertiary Hospital Using the Normalization Process Theory: Survey Study
Source: JMIR Hum Factors. 2023 Oct 6;10:e47390. doi: 10.2196/47390 (PMC10589830; doi:10.2196/47390)
Supplement: Multimedia Appendix 2 [file humanfactors_v10i1e47390_app2.pdf]

<u>Multimedia Appendix 2: NPT CORE CONSTRUCTS</u>

Coherence

Coherence is the sense-making work that people do individually and collectively when they are faced with the problem of operationalizing some set of practices. Like all NPT constructs it has four components.

1.1 Differentiation: An important element of sense-making work is to understand how a set of practices and their objects are different from each other. *For example, when doctors use a videoconferencing system to consult with patients, what do they do to understand and organise the differences between face-to-face consultations and videoconferencing.*
1.2 Communal specification: Sense-making relies on people working together to build a shared understanding of the aims, objectives, and expected benefits of a set of practices. *A great example is the team of investigators leading a clinical trial, as they work out how to integrate a complex clinical experiment into a healthcare setting, and as they try to identify and anticipate the relationship between elements of the trial and everyday clinical practice.*
1.3 Individual specification: Sense-making has an individual component too. Here participants in coherence work need to do things that will help them understand their specific tasks and responsibilities around a set of practices. *For example, nurses recruiting patients into a trial need to have a strong understanding of the work they must do to secure informed consent from patients, and how they will go about this.*
1.4 Internalisation: Finally, sense-making involves people in work that is about understanding the value, benefits, and importance of a set of practices. *So, returning to the example of doctors using a videoconferencing system to consult with their patients, it's about the work that they do to attribute worth to a new way of working.*

Cognitive Participation

Cognitive Participation is the relational work that people do to build and sustain a community of practice around a new technology or complex intervention. Like all NPT constructs, it has four components.

2.1 Initiation: When a set of practices is new or modified, a core problem is whether key participants are working to drive them forward. *For example, the work of setting up a clinical service is often delegated to a small group of managers and professionals who are charged with the work of setting up systems, procedures, and protocols and engaging with others to make things happen.*
2.2 Enrolment: Participants may need to organise or reorganise themselves and others to collectively contribute to the work involved in new practices. This is complex work that may involve rethinking individual and group relationships between people and things. *For example, getting nurses to 'buying in' to a falls prevention strategy is vital to its success,*

but the work of buying into the strategy is not simply about individual commitment, but is about building communal engagement.

2.3 Legitimation: An important component of relational work around participation is the work of ensuring that other participants believe it is right for them to be involved, and that they can make a valid contribution to it. *New service interventions are often founder because of a lack of investment in ensuring that they fit with the ways that different groups of professionals - and sometimes patients - define their possible contribution to them.*

2.4 Activation: Once it is underway, participants need to collectively define the actions and procedures needed to sustain a practice and to stay involved. *In fact, staying on the case is vital to sustaining clinical interventions. This is the work of keeping the new practices in view and connecting them with the people who need to be doing them*

Collective Action

Collective Action is the operational work that people do to enact a set of practices, whether these represent a new technology or complex healthcare intervention. Like all NPT constructs, it has four components. These were the first NPT constructs to be developed and their names reflect qualities of technologies or complex interventions, rather than the character of the work that these involve.

3.1 Interactional Workability: This refers to the interactional work that people do with each other, with artefacts, and with other elements of a set of practices, when they seek to operationalize them in everyday settings. *For example, a key problem of telemedicine systems has been shown to be their negotiation by doctors and patients as they try to communicate complex clinical information to each other over a videoconferencing link.*

3.2 Relational Integration: This refers to the knowledge work that people do to build accountability and maintain confidence in a set of practices and in each other as they use them. *A telemedicine system that transmitted clinical images of skin lesions ran into trouble when individual doctors began to lose confidence in what these images actually represented and started to examine patients in parallel to digitised images - thus doubling their workload and putting their clinical department under pressure.*

3.3 Skill set Workability: This refers to the allocation work that underpins the division of labour that is built up around a set of practices as they are operationalized in the real world. *Who gets to do the work is an important element of any set of practices. For example, a core problem for a research group investigating the effectiveness of a decision aid for medication choice after a serious illness event was whether the decision aid should be administered by trial managers with no clinical responsibility for the patient, or nurse practitioners actively involved in their care. Allocating the work to the former meant that the decision aid was more easily delivered, but trial managers lacked the clinical expertise of the nurse practitioners which meant that it was hard for them to answer patients' questions.*

3.4 Contextual Integration: This refers to resource work - managing a set of practices through the allocation of different kinds of resources and the execution of protocols, policies and procedures. *Typically, the implementation of a new set of practices is seen*

as a management problem, and it's true that the power to allocate resources and define the processes by which new technologies or complex interventions are executed in practice. The work that is involved in this is about resourcing the ways that others enact a new set of practices.

Reflexive Monitoring

Reflexive Monitoring is the appraisal work that people do to assess and understand the ways that a new set of practices affect them and others around them. Like all NPT constructs, it has four components:

4.1 Systematisation: participants in any set of practices may seek to determine how effective and useful it is for them and for others, and this involves the work of collecting information in a variety of ways. *The work of systematisation may be highly formal - the Randomised Controlled Clinical Trial is a prime example of formal systematisation. But it may also be very informal, the collection of anecdotal examples of problems in practice around a set of common themes by an unqualified care assistant is every bit as much an example of the systematisation of information.*
4.2 Communal appraisal: participants work together - sometimes in formal collaboratives, sometimes in informal groups to evaluate the worth of a set of practices. They may use many different means to do this drawing on a variety of experiential and systematised information. *These events happen continuously in almost every setting where people interact around a piece of hardware or new way of organising work and ask each other 'is it working?' How they put the answers to these questions and negotiate the difficulties that stem from conflicts about what sort of information counts, and how it counts for different groups, are central to the future of any set of practices. Acts of communal appraisal - like data analysis meetings in clinical trials, or quality circles in lean healthcare organisations - are common and may be highly formalised as well as casual and informal.*
4.3 Individual appraisal: Participants in a new set of practices also work experientially as individuals to appraise its effects on them and the contexts in which they are set. From this work stem actions through which individuals express their personal relationships to new technologies or complex interventions. *For example, a nurse working in a falls prevention program will work to appraise not only the worth of the program, but also its impact on her other tasks. So, a falls program that complicates and adds to an already complicated and demanding workload may well have a low value attributed to it in practice irrespective of its effects on falls within the hospital.*
4.4 Reconfiguration: appraisal work by individuals or groups may lead to attempts to redefine procedures or modify practices - and even to change the shape of a new technology itself. *For example, a nurse leading a falls prevention program might look again at the ways in which risk of falling was calculated in practice and the demands that this risk be placed on the delivery of nursing care elsewhere on the ward. If the work of calculating risk of falling was disproportionate to the work involved in dealing with other kinds of risks on the ward, then there would be pressure to modify the falls prevention program to make it workable in practice.*
